# Transcriptional precision in photoreceptor development and diseases – Lessons from 25 years of CRX research

**DOI:** 10.3389/fncel.2024.1347436

**Published:** 2024-02-13

**Authors:** Yiqiao Zheng, Shiming Chen

**Affiliations:** ^1^Molecular Genetics and Genomics Graduate Program, Division of Biological and Biomedical Sciences, Saint Louis, MO, United States; ^2^Department of Ophthalmology and Visual Sciences, Saint Louis, MO, United States; ^3^Department of Developmental Biology, Washington University in St. Louis, Saint Louis, MO, United States

**Keywords:** *CRX*, homeodomain, gene regulation, molecular genetics, inherited retinopathy, dominant diseases, pathogenic mechanisms

## Abstract

The vertebrate retina is made up of six specialized neuronal cell types and one glia that are generated from a common retinal progenitor. The development of these distinct cell types is programmed by transcription factors that regulate the expression of specific genes essential for cell fate specification and differentiation. Because of the complex nature of transcriptional regulation, understanding transcription factor functions in development and disease is challenging. Research on the *Cone-rod homeobox* transcription factor CRX provides an excellent model to address these challenges. In this review, we reflect on 25 years of mammalian CRX research and discuss recent progress in elucidating the distinct pathogenic mechanisms of four *CRX* coding variant classes. We highlight how *in vitro* biochemical studies of CRX protein functions facilitate understanding CRX regulatory principles in animal models. We conclude with a brief discussion of the emerging systems biology approaches that could accelerate precision medicine for *CRX*-linked diseases and beyond.

## Introduction

Photoreceptors are highly specialized cell types in the retina that “see” light. Light photons captured by photoreceptors are converted to electrical signals that travel through the optic nerve to the brain and form vision. In vertebrates, photoreceptors come in two major classes−rods and cones. The genesis and development of rods and cones follow a stereotypical order programmed by a photoreceptor gene regulatory network. This regulatory network also operates in adult retinas to ensure robust photoreceptor functions and cellular integrity. Components of this network and early events that regulate photoreceptor cell fate determination have been reviewed extensively (Swaroop et al., 2010; Bassett and Wallace, 2012; Brzezinski and Reh, 2015; Cepko, 2015; Wang and Cepko, 2016) and are not covered here. Rather, this review summarizes the mechanisms that regulate rod and cone differentiation after their fate is acquired through the lens of CRX studies in development and diseases. Most findings are based on the mammalian model organism *Mus musculus*, which provides the most comprehensive evidence on CRX protein functions.

The *Cone-rod homeobox* (*CRX*, OMIM: 602225, UniProt: O43186) gene encodes a homeodomain transcription factor that regulates gene expression programs essential for photoreceptor development, function, and maintenance. Coding variants in *CRX* have been associated with at least three types of retinopathies that result in blindness, including Leber Congenital Amaurosis (LCA), Cone-rod Dystrophies (CoRD), and Retinitis Pigmentosa (RP). To date, *CRX* is the only gene known to be associated with all three conditions, underscoring its critical role in both cone and rod biology. It is, therefore, important to understand CRX’s mechanisms of action in photoreceptors. Here, we review recent progress in elucidating CRX molecular functions in photoreceptor development and diseases. We highlight an integrated approach that draws on quantitative *in vitro* biochemical models, functional genomics in variant *knock-in* mouse retinas, and high-throughput screens built on systems biology principles. This holistic approach uncovers complex and intricate CRX regulatory principles that are otherwise elusive using conventional methodologies. These newly identified CRX regulatory principles explain the distinct pathogenic mechanisms in animal models, facilitate the functional predictions of other *CRX* coding sequence variants identified in clinical studies of *CRX*-linked diseases, and inform gene therapy development and precision medicine. We envision such an integrated approach is readily transferable to the study of transcription factors in other retinal cell types and their associated diseases.

## Identification of CRX as a master regulator of photoreceptor gene expression

**FIGURE 1:**
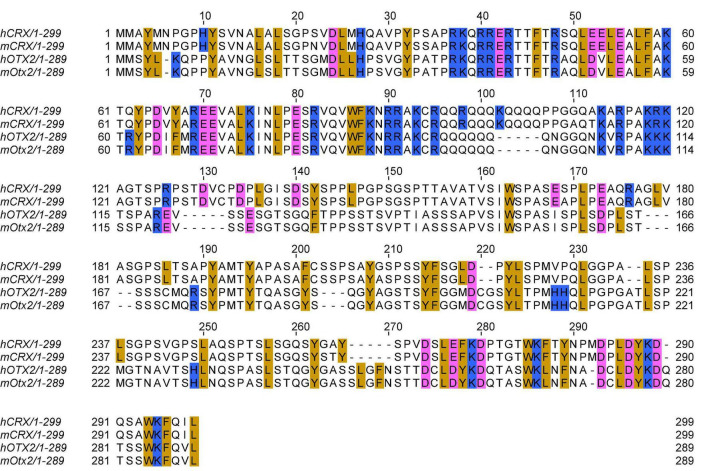
Multiple sequence alignment of human and mouse CRX and OTX2 protein sequences. Multiple sequence alignment is generated by the EMBL-EBI Clustal Omega program with default parameters. Selected amino acids are highlighted: golden: aromatic residues and Leucine; pink: acidic residues; blue: basic residues. Refseq protein sequence accession numbers: hCRX: NP_000545.1 (hg38); mCRX: NP_031796.1 (mml0); hOTX2: NP_068374.1 (hg38); mOTX2: NP_659090.1 (mml0).

**FIGURE 2:**
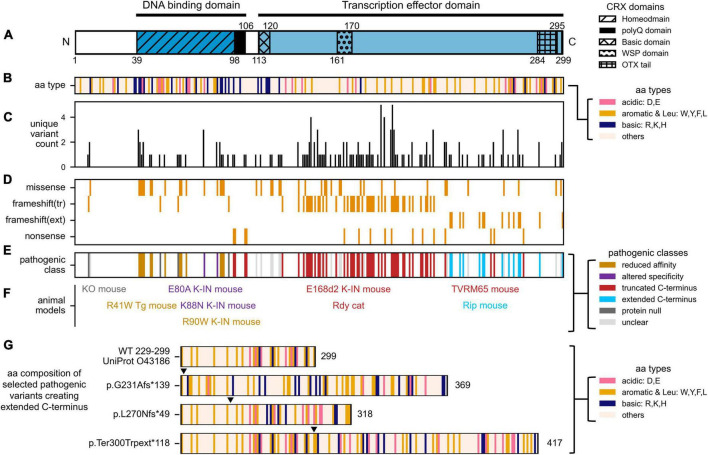
Distribution of human *CRX* coding variants and their predicted pathogenic classes. **(A)** Schematic showing full-length human CRX protein and its major domains, **(B)** Schematic showing the amino acid composition of full-length CRX protein. Acid, basic, aromatic, and Leucine residues are highlighted, **(C)** Bar chart showing the number of unique variants at each amino acid position, **(D)** Heatmap showing the type of protein sequence change reported at each amino acid position, **(E)** Diagram showing the predicted pathogenic classes based on the categorical approach. The complete list of curated *CRX* coding variants and accompanying references can be found in Supplementary Table 1. **(F)** Established animal models carrying different *Crx* variants. The labels are organized to match the relative amino acid positions of the *CRX* variants, and their colors match that of their pathogenic classes, **(G)** Diagrams comparing the amino acid composition of part of human CRX (UniProt: 043186) transcription effector domain (aa. 229–299) and selected variants predicted to create extended C-terminus with altered residue composition. Numbers accompanying each diagram represent the extended CRX protein length. Filled triangles above each diagram indicate the frameshift residue positions.

In 1997, three laboratories independently reported the cloning of the *CRX/mCrx* gene using complementary methods, including yeast one-hybrid system, cDNA hybridization, and degenerative RT-PCR (Chen et al., 1997; Freund et al., 1997; Furukawa et al., 1997). These studies demonstrated that *CRX/Crx* encodes a 299 amino acid sequence-specific DNA-binding protein, and it recognizes regulatory elements in the promoter of *rhodopsin*, a gene that encodes the rod-specific photopigment. The predicted human CRX and mouse CRX protein sequences only differ by 10 amino acids with 100% identity in the DNA binding domain (Figures 1, 2A). In addition, CRX protein shares sequence similarity in the DNA binding domain with many other homeobox family members implicated in early brain and eye development, including CHX10 (VSX2), OTX1, and OTX2 (Chen et al., 1997; Furukawa et al., 1997; Zheng et al., 2023). DNase I footprinting and transcription reporter assays identified CRX binding sites at photoreceptor gene regulatory sequences and demonstrated CRX’s primary function as a transcription activator. Multiple sequence alignment of the CRX bound and activated promoter sequences revealed an enriched DNA motif - CTAATC[C/T] – similar to that of the well-characterized *D. melanogaster* Bicoid homeodomain protein (Hanes and Brent, 1989, 1991). Protein truncation studies identified a C-terminus transcription effector domain (Figure 2A) responsible for CRX-mediated gene activation (Chau et al., 2000; Chen et al., 2002). Collectively, these early studies demonstrated that CRX is a homeodomain transcription factor that regulates photoreceptor gene expression and laid the foundation for CRX studies in animal models.

*In vivo*, *Crx*’s spatial and temporal expression patterns correlate with its roles in photoreceptor development and maintenance. In the mouse, cone genesis starts on embryonic day 10 (E10), and the last cones are born in the periphery on E18 (Carter-Dawson and Lavail, 1979; Young, 1985). Rod genesis partially overlaps that of cones, spanning from E13 to post-natal day 7 (P7). The peak of rod genesis is around the time of birth of the animal (P0). The expression of *Crx* transcripts is first detected at E12.5, localized to the outer aspect of the neural retina, corresponding to developing cones (Chen et al., 1997; Furukawa et al., 1997; Aavani et al., 2017). As cone genesis continues to increase after E12.5 and the initiation of rod genesis, the expression of *Crx* becomes stronger and remains restricted to the prospective photoreceptor layer. In the post-natal retina, *Crx* expression is observed throughout the prospective photoreceptor layer and reaches a peak at early post-natal ages (P6-7 in the *CD1* strain and P3-5 in the *C57BL/6J* strain). *Crx* expression then slightly decreases before settling at a high level maintained in mature rods and cones throughout adult life. It is important to note that these measurements are taken at the tissue level, and the slight expression drop after the peak might be a consequence of programmed cell death during normal development (Young, 1984; Braunger et al., 2014). *Crx* expression dynamics at a single cell level remains an important question to be addressed.

The disruption of CRX expression or function profoundly impacts photoreceptor development and survival. In the developing mouse retinas, retrovirus-mediated ectopic expression of *Crx* in P0 progenitor cells increases the number of rod photoreceptor-only clones, suggesting an instructive role of *Crx* in rod photoreceptor fate during retinal development (Furukawa et al., 1997). Genetic ablation of *Crx* in mice (*Crx-/-*) does not affect the genesis of photoreceptors but prevents their terminal differentiation and leads to rapid degeneration of the immature photoreceptor cells on or before P21 (Furukawa et al., 1999). CRX likely functions in cells that already adopt photoreceptor fate, where it promotes and maintains photoreceptor-specific gene expression programs. Indeed, lineage studies confirm that *Crx* expression is activated in post-mitotic photoreceptor precursors (Muranishi et al., 2011). Interestingly, ectopic expression of a dominant-negative form of CRX in P0 progenitor cells, with its homeodomain fused to the repressor domain of the *D. melanogaster* Engrailed protein, completely blocked rod terminal differentiation (Furukawa et al., 1997). Since the dominant negative CRX was ectopically expressed at a cell state when endogenous CRX is not activated, it might have perturbed additional programs not normally regulated by CRX and changed the intrinsic potentials of these cells to fully develop. Nevertheless, it emphasizes that photoreceptor development – and the underlying photoreceptor gene expression – is very sensitive to small quantitative differences in CRX regulatory activity. Mutations that either increase or decrease CRX activity can lead to diseases.

## CRX facilitates chromatin remodeling at photoreceptor regulatory regions

**FIGURE 3:**
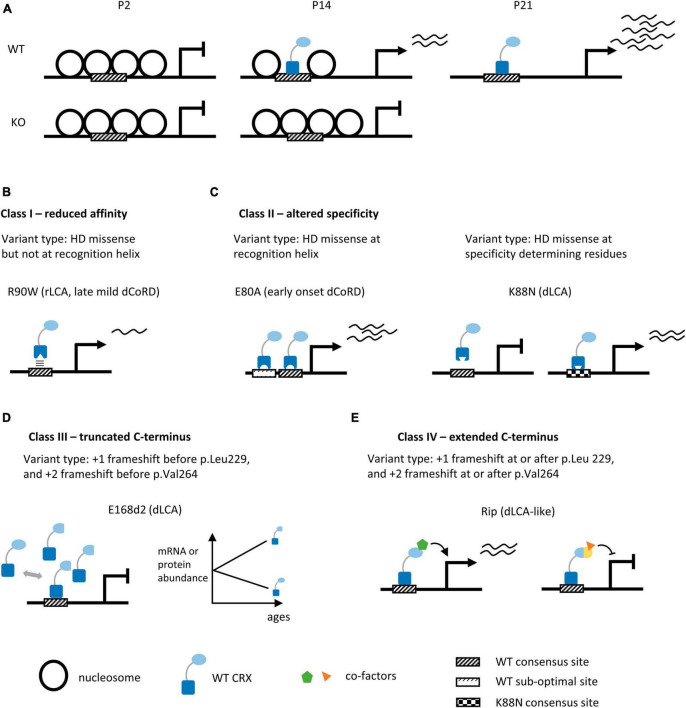
Summary of human CRX coding variants and their predicted pathogenic mechanisms in *knock-in* mouse models. **(A)** Diagrams depicting chromatin remodeling at CRX-dependent accessible sites in WT and *Crx* KO mouse retinas during post-natal development. P2, P14, P21: post-natal day 2, 14, 21. **(B–E)** Schematics highlighting the molecular mechanisms of human *CRX* coding variants in four pathogenic classes. We provide the type of variants for each class, the representative animal models, and associated phenotypes in humans, panels **(B,C)** depict models for missense variants of the CRX homeodomain (DNA binding domain), panels **(D,E)** depict models for frameshift and nonsense variants of the CRX transcription effector domain. +1 and +2 indicate the reading frame is shifted 1 or 2 bp 3′ to the original reading frame.

To understand CRX’s mechanisms of action *in vivo*, its DNA binding sites genome-wide were identified by chromatin immunoprecipitation followed by sequencing (ChIP-seq) in the developing mouse retinas (Corbo et al., 2010; Zheng et al., 2023). Recently, by comparing the high-resolution CRX ChIP-seq data from WT and *Crx* mutant retinas, Zheng et al. identified a set of “CRX-dependent genes” that rely on CRX binding at their regulatory elements for expression (Zheng et al., 2023). Many of the CRX-dependent genes are essential for photoreceptor structures and functions. The capacity of CRX to bind these regulatory elements also depends on the interactions with nucleosomes, which are structural units of the chromatin (Luger et al., 2012; Ahmad et al., 2022). Nucleosomes can inhibit the binding of many transcription factors, including CRX, by occluding their binding sites. Chromatin remodeling is a critical step to ensure transcription factors and transcriptional machinery have physical access to DNA. A comparison of the chromatin landscape in WT and *Crx* KO mouse retinas revealed that CRX is required for chromatin remodeling at a subset of its binding sites (Figure 3A; Ruzycki et al., 2018). During photoreceptor development, this subset of CRX binding sites increases accessibility and undergoes retinal-specific acquisition of epigenetic modifications associated with active promoters and enhancers, likely through CRX-dependent recruitment of chromatin remodeling complexes. DNA motif discovery analysis revealed that the CRX binding-dependent accessible sites tend to have a single enrichment of CRX consensus motifs while CRX-binding independent accessible sites have enrichment of additional neuronal transcriptional factor motifs. CRX likely adopts both independent and collaborative modes of action in different genomic contexts to regulate photoreceptor gene expression.

Although no chromatin remodeling defects were observed at CRX binding-independent accessible sites, the possibility of genetic compensation cannot be ruled out. The absence of *Crx* transcripts and/or proteins may activate the transcription of related genes, partially compensating for the loss of CRX. Accumulated evidence demonstrates that genetic compensation is a highly regulated process such that it is only triggered by certain types of genetic lesions (El-Brolosy and Stainier, 2017; El-Brolosy et al., 2019; Ma et al., 2019). A mutant *Crx* encoding a defective CRX protein that does not trigger such a compensation mechanism may affect the CRX-independent accessible sites and lead to more severe perturbations in photoreceptor differentiation than *Crx* KO animals. This model may explain why the loss of one *CRX* allele seems tolerated in heterozygous carriers while some *CRX* coding variants are associated with severe dominant phenotypes in humans.

## CRX interacts with other transcription (co-)factors to regulate photoreceptor gene expression

*Crx* is one of the earliest expressed photoreceptor-specific transcription factors, and its expression is essential for maintaining the expression of many downstream transcription factors and co-factors. It is important to note that in the *Crx* KO mouse retina, many of these downstream factors are expressed at early stages of photoreceptor development but diminish later, suggesting that initiation of their expression is independent of CRX, but their maintenance requires CRX. In addition to being a transcription activator, CRX also interacts – both directly and indirectly – with an array of transcription (co-)factors in stimulating photoreceptor gene expression (La Spada et al., 2001; Chen et al., 2004; Peng and Chen, 2005, 2007; Peng et al., 2005; Hennig et al., 2008; Onishi et al., 2009; Tomohiro et al., 2014; Andzelm et al., 2015). Extensive discussions on CRX and transcription (co-)factors in photoreceptor-specific gene regulation can be found in (Hennig et al., 2008; Swaroop et al., 2010). Here, we highlight two pairs of interactions – CRX-OTX2 and CRX-NRL – that are at the core of photoreceptor development.

### CRX-OTX2 division of labor at different stages of photoreceptor development

*Crx* and *Otx2* both belong to the *orthodenticle (otd)* gene family and encode homeodomain transcription factors that recognize a similar consensus DNA motif. The homeodomain sequence similarity (Figure 1) and the overlapping expression patterns in the developing mouse retinas led to the prediction that CRX and OTX2 function redundantly to regulate post-mitotic photoreceptor development (Chen et al., 1997; Furukawa et al., 1997). Targeted ablation of *Otx2* in the mouse retina using a transgenic *Crx-Cre* and ectopic OTX2 expression in the newborn mouse retinas suggest a modified model where OTX2 is involved in fixing newly post-mitotic cells to a committed photoreceptor precursor status and simultaneously upregulating *Crx* expression (Nishida et al., 2003; Koike et al., 2007; Wang S. et al., 2014). After the photoreceptor lineage is specified, CRX is responsible for terminal differentiation of the photoreceptors by inducing genes essential for cell-type specific functions. The downregulation of *Otx2* expression in post-natal photoreceptors and the concomitant upregulation of *Crx* expression also support this model (Wang S. et al., 2014). Yet, these pieces of evidence do not directly address the functional difference between CRX and OTX2 – is it due to the difference in the nature of the proteins or their different temporal expression patterns?

A recent mouse model study that heroically swapped the coding sequences of *Crx* and *Otx2* at their respective endogenous gene locus revealed that CRX and OTX2 functions are irreplaceable (Yamamoto et al., 2020). Specifically, insertion of *Crx* cDNA in the *Otx2* locus (*Otx2^Crx/Crx^*) leads to embryonic lethality as observed in the *Otx2*^–/–^ mice; conversely, replacement of *Otx2* cDNA in the *Crx* locus (*Crx*^*Otx*2/*Otx*2^) results in significant gene expression defects similar to that in the *Crx*^–/–^ retinas. This study unequivocally argues that CRX and OTX2 share some biochemical properties but have evolved distinct roles in regulating photoreceptor development *in vivo*. A related study in the *D. melanogaster* that compared the human CRX and OTX2 in their ability to rescue the retinogenesis defects in the *otd^uvi^* flies also found that CRX and OTX2 each mediated a defined subset of *otd*-dependent functions (Terrell et al., 2012). Mechanistically, many questions remain – How are the opposite post-natal expression dynamics of *Crx* and *Otx2* in post-mitotic photoreceptors regulated? What is the molecular basis for the distinct functions of CRX vs. OTX2? How do local genomic contexts and/or interacting (co-)factors modulate CRX vs. OTX2 activity at distinct and overlapping regulatory regions?

### CRX-NRL synergistic activation of the *rhodopsin* promoter *in vitro*

CRX-NRL-mediated synergistic activation of the *rhodopsin* promoter is another prominent example of CRX-transcription factor interactions. NRL is a basic leucine zipper (bZIP) transcription factor specifically expressed in rods (Chen et al., 1997). The synergistic activity requires the simultaneous presence of CRX and NRL with intact DNA binding domains (DBDs) and DNA motifs for both factors (Mitton et al., 2000). The co-occupancy of CRX and NRL binding sites likely induces deformation of the DNA template and generates a favorable interface for general transcriptional machinery (Van der Vliet and Verrijzer, 1993). Subsequent studies found that other homeodomain proteins that bind CRX DNA motifs can also act synergistically with NRL at the *rhodopsin* promoter (Onorati et al., 2007; Reks et al., 2014). This raises the possibility that the activity synergy is a shared property of homeodomain family proteins and members from the bZIP superfamily, which includes the JUN, FOS, ATF, and MAF families that control fundamental cellular processes, including cell proliferation, differentiation, and apoptosis. Based on this model, CRX regulates not only genes for photoreceptor-specific structures and functions but also essential genes that control basic cellular functions.

Despite the dramatic activity synergy observed in cell culture reporter assays, the functional importance of CRX-NRL synergy *in vivo* remains elusive. Additionally, the *rhodopsin* promoter contains many motifs bound by other transcription factor families – a feature not shared by many other photoreceptor genes. These raise some important questions – What is the extent of CRX-NRL synergy in regulating rod photoreceptor genes? How is CRX-NRL synergistic activation different from simple additive activation? How are the genes/promoters dependent on CRX-NRL synergy for activation different from those that are not?

In contrast to rod genes, CRX regulation of cone genes *in vivo* is less understood except for its expression in cone cells. Cell line reporter assays demonstrate similar transcriptional activator activity of CRX on cone-specific gene promoters, including *arrestin*, *opn1sw*, and *opn1mw* (Chen et al., 1997; Irie et al., 2015; Zheng et al., 2023). It is popularly believed that CRX binds the same DNA motif in regulating rod and cone gene expression. Since cones are born at a distinct period, from a different pool of retinal progenitor cells than rods (Nakamura et al., 2006; Shibasaki et al., 2007; Hafler et al., 2012), cones are probably intrinsically different from rods both in their epigenetic characteristics and the repertoire of transcription factors at their disposal (Forrest and Swaroop, 2012; Emerson et al., 2013; Sapkota et al., 2014; Jean-Charles et al., 2018). Thus, a pathogenic *CRX* variant may have distinct impacts on the development, function, and survival of cones vs. rods.

## Disease-associated *CRX* coding variants

In the most recent ClinVar release, 338 *CRX* coding variants have been documented – 80 annotated as pathogenic/likely pathogenic, 192 as uncertain significance/conflicting, 77 as benign/likely benign, and 11 as other. *CRX* coding variants are associated with at least three forms of inherited retinal disorders (IRDs) that cause blindness, including Leber congenital amaurosis 7 (LCA7, OMIM: 613829), Cone-rod dystrophy 2 (CoRD2, OMIM: 120970), retinitis pigmentosa (RP, OMIM: 268000). Figures 2C, D summarize our curated *CRX* coding variants identified in individuals with vision problems. Figure 2C presents the number of unique *CRX* variants at each amino acid position, and Figure 2D shows the type of protein sequence change at each position. A complete list of our curated set of *CRX* coding variants and accompanying references can be found in Supplementary Table 1.

*CRX*-linked retinopathies vary greatly in the age of onset, rate of progression, and severity, reflecting the complexity of CRX’s mechanisms of action and highlighting challenges in evaluating sequence variants in different genetic backgrounds. Despite heterogeneity in clinical phenotypes, most *CRX* coding variants arise *de novo*, appear completely penetrant, and cause diseases in heterozygotes (autosomal dominant). Multiple reports have described putative *CRX* null variants to be tolerated in heterozygous carriers or are associated with variable phenotypes in the family, preventing a conclusive genotype-phenotype correlation from being drawn (Silva et al., 2000; Jones et al., 2017; Ibrahim et al., 2018; Yahya et al., 2023). These patterns suggest that haploinsufficiency is not the key mechanism of pathogenesis for dominant *CRX* variants. Consistently, heterozygous deletion of *Crx* only produces very mild phenotypes in the *Crx^+/–^* mouse retinas (Furukawa et al., 1999). Therefore, the *Crx* KO mouse does not provide an appropriate model for severe dominant *CRX* diseases.

To study *CRX* disease variants effectively, a categorical approach has been employed (Tran and Chen, 2014). In this paradigm, disease variants are categorized into four major classes based on their locations in CRX functional domains and the impacts on CRX biochemical properties (Figure 2E). For each class, one or more representative human variant knock-in mouse models (Figure 2F) were created and subjected to in-depth molecular and cellular characterizations (Supplementary Table 2). This approach has yielded invaluable insights into different pathogenic mechanisms, both developmental and degenerative, revealed the multifaceted roles CRX plays in regulating photoreceptor biology, and laid the foundation for developing targeted gene therapies against different disease mechanisms. In the following four sections, we briefly describe the major findings from mouse models of different pathogenic classes and discuss the lessons learned on CRX functions during normal development.

### Frameshift and non-sense variants produce truncated CRX effector domain

Indels – insertions and deletions – that create frameshift and/or pre-mature termination of *CRX* translation are concentrated in the C-terminus CRX transcription effector domain (Figures 2A, D). These variants are predicted to produce truncated CRX proteins that retain a full-length DNA binding domain and intact DNA binding activity but are defective in CRX-mediated gene activation. *Crx*^*E*168*d*2^ knock-in mouse (E168d2) is a representative model for variants that produce truncated CRX C-terminus (Tran and Chen, 2014; Tran et al., 2014). In humans, E168d2 [NM_000554.6:*c.503_504del* (p.Glu168fs), ClinVar VCV000099609] is associated with dominant LCA (Freund et al., 1998; Jacobson et al., 1998). The *E168d2/+* mouse retinas have 6–8 rows of photoreceptor nuclei left in the outer nuclear layer (ONL) by 3mo, and *E168d2/d2* retinas have only 3–4 rows left by 1mo (Supplementary Table 2). *E168d2/+* mice have no detectable cone function and severely impaired rod function at 1 month, followed by a complete loss of rod function by 3 months. *E168d2/d2* mice never develop any visual function. Molecular studies in the *E168d2/+* mouse retinas reveal an allelic-specific overexpression of the mutant transcripts and accumulation of the non-functional, truncated proteins, resulting in a significantly elevated mutant-to-WT ratio (Figure 3D). The impaired photoreceptor differentiation in the *E168d2/+* retinas can thus be explained by a dominant-negative mechanism where the significantly higher concentrations of the mutant proteins outcompete WT proteins in binding to CRX cognate sites and interfere with downstream gene regulations. A similar allelic-specific overexpression mechanism has also been reported in *Crx^Rdy^* cat, the earliest documented animal model carrying a truncating mutation in the CRX effector domain (Menotti-Raymond et al., 2010a,b; Occelli et al., 2016; Occelli et al., 2023).

Subsequent studies on variants under the same class revealed a positive correlation between the CRX C-terminus truncation length and the degree of allelic-specific imbalanced expression and the onset of photoreceptor degeneration in animal models (Supplementary Table 2) (Tran et al., 2014; Ruzycki et al., 2015, 2017). This suggests the existence of multiple regulatory signals in the mRNA sequences encoding CRX transcription effector domain, which are exposed by pre-mature translation termination (PTC), and these signals act additively to stabilize the mutant *Crx* transcript ectopically. The Exon Junction Complex (EJC) model of mammalian non-sense mediated decay (NMD) suggests that transcripts with PTCs in the 3’ portion of the gene, including the last exon and ∼55 bp of the penultimate exon, are typically stably translated into truncated proteins (Khajavi et al., 2006). Since the entire CRX transcription effector domain is encoded by sequences in the last exon of *CRX*, allelic truncating mutations in the CRX transcription effector domain likely produce mutant mRNA that escapes NMD. Such an “escape from NMD surveillance” mechanism has been proposed to modulate the ultimate phenotype for multiple human diseases associated with a single disease gene conveying phenotypes that segregate as dominant *versus* recessive traits (Khajavi et al., 2006). Yet, other models suggest that the long 3’-UTRs created by PTCs can trigger NMD by promoting the binding of a central regulator of NMD (He and Jacobson, 2015). Thus, elucidation of the molecular mechanisms of CRX effector domain truncating mutations will advance our understanding not only of *CRX*-linked retinal diseases but also of more general cellular processes such as mRNA surveillance pathways.

### Frameshift variants produce extended non-homologous CRX effector domain

Opposite to the first class, frameshift variants can also produce an elongated mutant CRX protein with a partial transcription effector domain and a non-homologous extension in the C-terminus. *Crx^Rip^* mice (Rip: Retina with Immature Photoreceptors, MGI:5515375) carrying a spontaneous 1-bp deletion in *Crx* exon 4 [NM_007770.4: *c.763del* (p.Gly255Alafs*133)], is a model for variants in this class (Roger et al., 2014). Distinct from the rapid photoreceptor degeneration phenotype in *E168d2* models, the thickness of ONL in the *Rip/+* retina is largely preserved for at least up to 18 months, even though the *Rip/+* mouse is completely blind at 1 month (Supplementary Table 2). This suggests that defective expression of photoreceptor genes and/or incomplete differentiation is not a sufficient signal to trigger photoreceptor degeneration. The *Crx Rip* transcript is not overproduced in the mutant mouse retinas, likely because the entire *Crx* coding mRNA sequences are still translated. It has been proposed that removal of the OTX tail domain (CRX aa.284-296, Figure 2A) in the CRX RIP protein disrupts the recruitment of WT CRX and OTX2 and thus reduces the expression of downstream transcriptional regulators during photoreceptor development. Since the E168d2 mutant protein also lacks the OTX tail domain, further experiments are needed to explain why *Rip/+* retina is associated with more severe functional deficits than *+/-* and how CRX RIP protein antagonizes WT CRX functions in regulating photoreceptor gene expression.

In recent years, it is gradually appreciated that the function of transcription effector domain – activation, repression, or both – relates not to the exact amino acid sequences but instead to the composition and patterning of charged and hydrophobic residues (Boija et al., 2018; Staller et al., 2018, 2022; Sanborn et al., 2021; DelRosso et al., 2023; Kotha and Staller, 2023). In general, strong activator activity requires a balance of acidic, aromatic, and leucine residues – which is observed toward the C-terminus end of CRX (Figures 1, 2A, B). The amino acid composition of the transcription effector domain also determines the selective recruitment of transcriptional co-factors, mediators, and histone binding complexes. Therefore, the addition of the 133 amino acids in the CRX RIP protein, and similarly for other variants that produce extended C-terminus, may disrupt WT CRX effector domain residue patterning (Figure 2G), which could impact the affinity and/or specificity of recruiting transcription (co-)factors and mediators beyond just OTX2 and CRX and result in dramatic gene misregulation genome-wide (Figure 3E).

Collectively, at least two pathogenic mechanisms of CRX effector domain variants exist. Truncating variants are associated with over-expression of the transcriptionally incompetent mutant CRX proteins that likely out-compete WT CRX in binding to photoreceptor regulatory sequences and consequently perturbing gene activation during development. Elongating variants produce mutant CRX with an extended effector domain with altered amino acid compositions that likely perturb the recruitment of regulatory proteins that collaborate with CRX in regulating photoreceptor genes.

### Missense variants reduce CRX homeodomain DNA binding affinity

Unlike variants in the transcription effector domain, disease variants in the CRX homeodomain are predominantly single amino acid substitutions (Figures 2A, D). Homeodomain is a 60 amino acid helix-turn-helix (HTH) DNA binding domain present in a large and diverse group of proteins that play indispensable roles in embryonic development (Mark et al., 1997; Banerjee-Basu and Baxevanis, 2001; Bürglin and Affolter, 2016). Despite variability in amino acid sequences, the 3-dimensional structure and modes of DNA contacts are conserved in different subfamilies of homeodomain proteins and in different organisms (Gehring et al., 1990, 1994a; 1994b). Multiple structures of *paired* class homeodomains bound to their respective consensus DNA sequences have been solved (Otting et al., 1990; Güntert et al., 1991; Billeter et al., 1993; Fraenkel and Pabo, 1998; Chaney et al., 2005; Baird-Titus et al., 2006). The wealth of information on homeodomain molecular properties has been invaluable to the recent discovery of novel gain-of-function pathogenic mechanisms of CRX homeodomain missense variants.

CRX-mediated gene activation depends on its binding to cognate DNA sequences. One apparent pathogenic mechanism of CRX homeodomain variants is to reduce CRX’s binding affinity to DNA. *Crx*^*R*90*W*^ is a representative model for the hypomorphic class variants that reduce CRX’s DNA binding affinity (Tran et al., 2014). R90W variant [NM_000554.6: *c.268C > T* (p.Arg90Trp), ClinVar VCV000007422] is associated with recessive LCA and mild late-onset dominant CoRD (Swaroop et al., 1999; Fujinami-Yokokawa et al., 2020; Ng et al., 2020). Biochemical assays found that R90W HD has significantly reduced DNA binding affinity and activates photoreceptor gene promoters poorly (Chen et al., 2002; Figure 3B). As expected, the *Crx*^*R*90*W/W*^ mouse shows photoreceptor degeneration phenotypes similar to that observed in *Crx*^–/–^ (Supplementary Table 2).

Patient-specific variants at R40(HD2), R41(HD3), and R43(HD5) residues at the homeodomain N-terminus also significantly reduce CRX’s DNA binding affinity (Chen et al., 2002). Some of these variants are associated with more severe dominant retinal dystrophies (Supplementary Table 1). Based on structural studies, CRX R90 (HD52) is not involved in direct DNA contact and instead helps to stabilize the HD-DNA binding structure through intramolecular interactions with other homeodomain residues (Chaney et al., 2005; Baird-Titus et al., 2006). Different from R90, homeodomain N-terminus residues make specific contacts with DNA bases in the minor groove. These interactions are essential for the recognition of the 5′-TAAT-3′ DNA core motif, a key property for homeoprotein DNA binding (Noyes et al., 2008; Chu et al., 2012). The vital structural functions provide an explanation for the prevalence of R40-R43 variants in individuals with severe dominant vision problems (Figure 2C).

A typical pattern of CRX missense variants affecting DNA binding affinity is substituting a conserved charged residue with a neutral or a hydrophobic residue, such as R > Q and R > W. Intuitively, these substitutions change CRX DNA binding strength, correlatively reducing CRX’s transactivation activity and perturbing the highly coordinated developmental programs. Thus, the severity of disease phenotypes in this class can be largely predicted based on the mutant CRX DNA binding affinity deviation from the WT CRX protein.

### Missense variants alter CRX homeodomain DNA binding specificity

The other class of CRX homeodomain missense variants perturb CRX’s DNA binding specificity. Unlike the simplest model – each transcription factor binds one consensus sequence – most transcription factors bind to degenerative sequences that harbor nucleotide variants from the consensus. The DNA binding specificity characterizes a transcription factor’s preference or relative binding affinities at such a collection of degenerative DNA motifs (Stormo and Zhao, 2010; Stormo, 2013). A recent study elucidated two novel gain-of-function mechanisms for variants, p.E80A and p.K88N, that alter CRX DNA binding specificity differently (Zheng et al., 2023).

*Crx*^*E*80*A*^ (E80A) is a model for missense variants that preserve CRX’s DNA binding preference but reduce the overall “selectivity” in binding. E80A [NM_000554.6:*c.239A > C* (p.Glu80Ala), ClinVar VCV000007416] is associated with severe early-onset dominant CoRD in humans (Hittner et al., 1975; Freund et al., 1997; Sohocki et al., 1998, 2001). E80A knock-in mouse models recapitulate human phenotypes – the *E80A/+* mouse has no detectable cone-mediated light responses and is defective in rod-mediated light responses at 1 month (Supplementary Table 2). Albeit disorganized ONL structures and shortened photoreceptor outer segments (OS), no obvious photoreceptor degeneration is observed in these retinas. Both *in vitro* and *in vivo* assays show that CRX E80A binds WT CRX cognate sites and drives elevated expression of target genes in early photoreceptor development. Biochemical evidence suggests that the E > A substitution, which changes a negatively charged residue to a small hydrophobic residue, likely results in a gain of entropy such that the mutant CRX E80A protein binds more promiscuously to non-consensus/sub-optimal CRX motifs and drives a higher level of gene expression (Figure 3C; Wilson et al., 1995; Chaney et al., 2005). Coordinating different cellular programs is essential to build a functional tissue. CRX E80A mediated hyper gene activation in early development may lead to asynchronization of CRX-regulated processes from other developmental programs, affecting photoreceptor terminal differentiation. Importantly, variants at E80 residue are all associated with severe early-onset dominant CoRD in humans (Supplementary Table 1) – it remains to be understood why cones are more sensitive to E80 variants than rods – are cones intrinsically more prone to any perturbations? Or is CRX E80 residue specifically more critical for cone gene regulation?

*Crx*^*K*88*N*^ (K88N) is a model for missense variants that specifically affect the homeodomain specificity determining residues. K88N [NM_000554.6:*c.264G > T* (p.Lys88Asn), no ClinVar entry] is associated with severe dominant LCA in humans (Nichols et al., 2010). Although not evidently degenerating, the *K88N/+* and *K88N/N* mice show more severely disturbed retinal morphology than *Crx* KO or *R90W/W* and are completely blind at 1 month (Supplementary Table 2). Since the *+/-* and *R90W/+* mouse retinas are morphologically normal and functionally intact at 1 month, it suggests that changing CRX DNA binding specificity is more deleterious than simply losing WT CRX functions. High-throughput *in vitro* DNA binding specificity assay, Spec-seq, reveals that K88N mutation changes CRX preferred sequence from TAATCC to TAATT[A/T]. CRX K88N *in vivo* binding at WT cognate sites is diminished with a concomitant binding enrichment at novel sites enriched for the Spec-seq found N88 motifs (Figure 3C). Compared to the hypomorphic model *R90W/W*, *K88N/N* retinas show greater gene expression loss and more severe photoreceptor developmental deficits. Even in *K88N/+* retinas, most photoreceptor-specific genes remain severely under-expressed at P21, suggesting that the ectopic CRX K88N activity functionally antagonizes CRX WT’s actions. The N88 homeodomain preferred DNA motif resembles that of other important retinal transcription factors, including RAX, VSX2/1, and LHX family members. Therefore, it is likely that CRX K88N may even interfere with other transcription factor regulatory activities by ectopically binding to a subset of their cognate sites. The exact molecular mechanisms await further investigations.

DNA binding specificity is one important mechanism that transcription factors evolve to achieve functional specificity such that genes with even a slight difference in DNA motif in their regulatory regions can respond differently to changing compositions of transcription factors and drive distinct phenotypic outcomes during development. One remarkable example is an 11bp activator homeodomain motif, either in the palindromic version, regulates phototransduction genes that are expressed broadly in all photoreceptors; or when exhibiting unique single base pair substitutions, restrict *D. melanogaster rhodopsin* genes to be expressed in subsets of photoreceptors (Rister et al., 2015; Poupault et al., 2021; Datta and Rister, 2022). These seemingly “trivial” differences in the 11bp palindromic motifs coordinate a broad spectrum of homeodomain proteins of different DNA binding specificity and transactivation activity in the developing photoreceptors (Tahayato et al., 2003; Mishra et al., 2010; Johnston et al., 2011). Therefore, variants affecting homeodomain DNA binding specificity are expected to lead to more severe phenotypic outcomes than variants that simply reduce CRX’s DNA binding affinity. Indeed, the CRX homeodomain recognition helix – CRX residues 80–93 – are populated by disease variants, many of which are associated with dominant LCA, emphasizing the importance of precise CRX-DNA interactions in regulating photoreceptor normal development and functions.

In summary, at least two pathogenic mechanisms exist for CRX homeodomain variants. Hypomorphic variants that perturb homeodomain-DNA binding complex stability reduce CRX’s binding affinity at its cognate sequences and lead to down-regulation of CRX target gene expression. Antimorphic variants that lower or alter CRX’s DNA binding specificity divert mutant CRX to non-cognate sequences and result in either precocious target gene activation or ectopic regulatory activities that antagonize WT CRX functions, both leading to severe dominant phenotypes that are distinct from that caused by hypomorphic variants.

## Systematic prediction of *CRX* coding variants

Once we understand how CRX regulates gene expression in normal conditions and representative variant knock-in animal models, we can make functional predictions of newly identified coding variants. Instead of testing one variant at a time, deep mutational scanning (DMS) is an emerging strategy to efficiently assay the functional consequences of hundreds to thousands of different protein variants in parallel (Fowler and Fields, 2014). The development of machine learning methods in protein structure prediction and pattern discovery has also significantly reduced the barriers to comprehending the enormous amount of data generated by typical DMS experiments. With deep mutational scanning, we can generate a lookup table of all possible single amino acid substitutions in CRX and even combinatory variants. We can then build on the categorical approach and associate every uncharacterized variant to one of the characterized variants by their similarity in affecting CRX’s intrinsic properties. Insights gained from *Crx* animal models will guide the design of DMS experiments, for example, by selecting the most biologically meaningful readouts for variants in different pathogenic classes.

## From coding to non-coding variants at CRX bound regulatory regions

Coding variants in CRX homeodomain impact CRX DNA-binding interactions and perturb target gene expression. Similarly, non-coding variants that change CRX DNA binding site sequences could perturb CRX target gene expression. Interactions between *CRX* coding variants and CRX binding site non-coding variants constitute another layer of complexity and may account for the missing heritability and phenotypic heterogeneity of a subset of disease-linked *CRX* coding variants (Zhang and Lupski, 2015). Since perturbation of many CRX target genes alone can lead to severe vision problems, understanding CRX regulatory functions globally and at the gene-specific level are both necessary. A comprehensive and quantitative CRX model built on in-depth animal studies and systems biology principles should fulfill such a need, considering both CRX intrinsic activities and additional factors, such as fluctuations of CRX protein levels, interactions with other factors, and the chromatin environment. One example is the application of Massively Parallel Reporter Assays (MPRAs) to assess the regulatory activities of CRX-bound genomic sequences in *ex plant* WT and disease variant knock-in mouse retinas (White et al., 2016; Hughes et al., 2018; Friedman et al., 2021; James et al., 2023). When combined with rationally designed mutagenesis libraries, one can start to understand the importance of CRX in different genomic contexts and predict the degree of impact on different genes in response to mutant CRX activity. This information could help prioritize non-coding variants−whether it protects or sensitizes a gene to *CRX* coding variants. Besides MPRAs, many assays have been developed to interrogate other aspects of transcription factor-gene expression relationships and can be readily adapted to the retinal system (Wang et al., 2012; Arnold et al., 2013; Dixit et al., 2016; Muraro et al., 2016; Maricque et al., 2019).

## Toward *CRX* gene therapy

As a master transcriptional regulator, CRX controls many aspects of photoreceptor biology. It can be more challenging to treat diseases caused by variants in *CRX* than in a gene with a discrete function. With many years of studies on CRX *in vitro* and in animal models, we are now starting to explore different therapeutic strategies to target different pathogenic mechanisms. For example, supplementing the normal gene – known as gene-augmentation therapy – may be sufficient to treat loss-of-function variants, while simultaneous silencing or removal of the mutant allele will be critical for treating antimorphic variants. To the extent of data published, at least four groups have attempted proof-of-concept strategies both in animal models (Roger et al., 2022; Sun and Chen, 2023) and in organoid models (Chirco et al., 2021; Kruczek et al., 2021) targeting mutations in all four pathogenic classes. To advance gene therapy to treat *CRX*-associated disorders, there are many important questions await careful evaluation, such as developmental vs. degenerative pathogenic mechanism, effective treatment window, toxicity of CRX overexpression, and neuroplasticity of diseases photoreceptor cells (Sun and Chen, 2023). Nevertheless, these early studies demonstrate that, with careful design, gene therapy could be a viable strategy for *CRX-*associated diseases.

## Future directions

From 25 years of CRX research, we now know that photoreceptor gene regulation is a highly coordinated process that requires fine-tuned CRX transcription factor activities. Expanded from the simplest model – CRX binds its consensus motif 5′-TAATCC-3′ and activates gene expression, it is now clear that CRX DNA binding affinity and specificity, interaction with collaborating transcription (co-)factors, and interplay with local and broader chromatin environment collectively contribute to the precise gene expression programs that constitute the molecular foundations of photoreceptor development, functions, and long-term survival. Variants that differentially disrupt CRX activities lead to different disease manifestations, underscoring the intricate connections of CRX functions in various aspects of photoreceptor biology.

These observations also open new research avenues of CRX functions and photoreceptor biology. For example, a large subset of *CRX* disease mutations is associated with severe early-onset dominant CoRD (e.g., E80A), suggesting cone and rod photoreceptors may rely on different CRX regulatory principles or have different degrees of dependency on CRX activity. For example, cone genes and rod genes may rely on different types of homeodomain motifs that are differentially bound by CRX WT and CRX E80A. The limited cone population in the mouse retina has been prohibitive to high-throughput quantitative studies (Jeon et al., 1998). A cone-dominated retina (chicken and ground squirrel) will be better suited to tackle this question. Relatedly, mutations in the same *CRX* variant class can be associated with progressively worsened phenotypes in knock-in mouse models, pointing to a quantitative connection between CRX transcription factor functions and the sensitivity/resilience of different CRX-regulated genes. For instance, a gene controlled by multiple copies of consensus CRX motifs is more likely to buffer against fluctuations in CRX activity than a gene regulated by sub-optimal CRX motifs. A systematic comparison between different *Crx* animal models may offer important insights into this model. Lastly, proteins involved in DNA sequence-independent interactions with CRX are a less explored area. As discussed above, the heterogeneity of CRX transcription effector domain variants may be attributed to differential impacts on CRX interacting factors. High-throughput, quantitative systems that combine proximity labeling and mass spectrometry are now available to answer these questions (Roux et al., 2012; Rhee et al., 2013; Lam et al., 2015; Kim et al., 2016; Schopp et al., 2017; Branon et al., 2018; Ramanathan et al., 2018).

In the past two decades, the dramatic increase in genetic testing has led to the identification of many coding variations in transcription factors important for retinal development and homeostasis. To this date, it remains a significant challenge to identify specific disease-causing variants and to make an accurate prognosis. We believe the integrated approach of CRX research provides one solution to these challenges and will accelerate the development of personalized medicine for rare genetic diseases affecting the retina and other tissues.

## Author contributions

YZ: Conceptualization, Data curation, Visualization, Writing–original draft, Writing–review and editing; SC: Conceptualization, Funding acquisition, Writing–review and editing.
