# Supplementary material for: Transcriptional precision in photoreceptor development and diseases – Lessons from 25 years of CRX research
Source: Front Cell Neurosci. 2024 Feb 13;18:1347436. doi: 10.3389/fncel.2024.1347436 (PMC10896975; doi:10.3389/fncel.2024.1347436)
Supplement: Supplementary file 1 [file Table_1.docx]

| Supplementary Table S1 Documented *CRX* coding variants – related to Figures 2c and 2d  * Genomic coordinates are given in GRCh38 (hg19) assembly if not otherwise specified. For one variant - p.D219fs – the genomic coordinate is not reported in the original publication and labeled as unavailable.  ** ClinVar accession number is only available for variants with a ClinVar entry.  † ad: autosomal dominant; ar: autosomal recessive; LCA: Leber congenital amaurosis; CoRD: Cone rod dystrophy; RP: retinitis pigmentosa; RD: retinal dystrophy | | | | | |
| --- | --- | --- | --- | --- | --- |
| **Allele name*** | **Reported inheritance†** | **Reported condition†** | **ClinVar accession**** | **PubMed records** | **Reference** |
| NM_000554.6(CRX):c.24dup (p.Pro9fs) | ad/ar | LCA, but inconsistent in family | VCV000099598 | PMID: 10892846 | (Silva et al., 2000) |
| NM_000554.6(CRX):c.28C>G (p.His10Asp) | ad | RP | VCV000329693 | PMID: 11139241, 30718709 | (Sohocki et al., 2001, Jespersgaard et al., 2019) |
| NM_000554.6(CRX):c.29A>G (p.His10Arg) | ad | Usher | VCV000981463 |  |  |
| NM_000554.6(CRX):c.118C>T (p.Arg40Trp) | ad | CoRD, RCD | VCV000864383 | PMID: 26161267, 28041643, 29785639, 31626798, 32533067, 33546218, 37239417 | (Arai et al., 2015, Carss et al., 2017, Maeda et al., 2018, Yi et al., 2019, Fujinami-Yokokawa et al., 2020, Maggi et al., 2021, Kim et al., 2023) |
| NM_000554.6(CRX):c.119G>C (p.Arg40Pro) | ad | CoRD | VCV002037356 | PMID: 26161267 | (Arai et al., 2015) |
| NM_000554.6(CRX):c.119G>A (p.Arg40Gln) | ad | CoRD | VCV000437959 | PMID: 9792858, 10892846, 10916183, 26910043, 28041643 | (Sohocki et al., 1998, Silva et al., 2000, Tzekov et al., 2000, Blanco-Kelly et al., 2016, Carss et al., 2017) |
| NM_000554.6(CRX):c.121C>T (p.Arg41Trp) | ad | CoRD | VCV000007418 | PMID: 9427255, 10916183, 11748859, 12819982, 26957898, 27208204, 31626798, 32533067, 36909829, 37239417 | (Swain et al., 1997, Tzekov et al., 2000, Rivolta et al., 2001b, Itabashi et al., 2003, Oishi et al., 2016, Ellingford et al., 2016a, Yi et al., 2019, Fujinami-Yokokawa et al., 2020, Peter et al., 2023, Kim et al., 2023) |
| NM_000554.6(CRX):c.122G>A (p.Arg41Gln) | ad | CoRD | VCV000007421 | PMID: 9427255, 9792858, 11748859, 26103963, 29847639, 31215831, 32165824 | (Swain et al., 1997, Sohocki et al., 1998, Rivolta et al., 2001b, Boulanger-Scemama et al., 2015, Martin-Merida et al., 2018, Chapi et al., 2019, Surl et al., 2020) |
| NM_000554.6(CRX):c.124G>A (p.Glu42Lys) | ad | LCA | VCV000216914 | PMID: 21602930, 31630094 | (Li et al., 2011, Xu et al., 2020) |
| NM_000554.6(CRX):c.127C>T (p.Arg43Cys) | ad | CoRD | VCV000636019 | PMID:  25270190, 30718709, 31626798, 32533067 | (Hull et al., 2014, Jespersgaard et al., 2019, Yi et al., 2019, Fujinami-Yokokawa et al., 2020) |
| NM_000554.6(CRX):c.128G>A (p.Arg43His) | ad | MD, LCA | VCV000956064 | PMID: 31626798, 32533067, 37239417 | (Yi et al., 2019, Fujinami-Yokokawa et al., 2020, Kim et al., 2023) |
| NM_000554.6(CRX):c.139A>C (p.Thr47Pro) |  | RP | VCV000931935 |  |  |
| NM_000554.6(CRX):c.142C>T (p.Arg48Trp) | ad | CoRD | VCV001400605 | PMID: 26957898 | (Oishi et al., 2016) |
| NM_000554.6(CRX):c.159del (p.Asn53fs) | ad | CoRD |  | PMID: 29555955 | (Birtel et al., 2018) |
| NM_000554.6(CRX):c.166G>A (p.Ala56Thr) | ad | LCA | VCV000099596 | PMID: 10766140 | (Lotery et al., 2000) |
| NM_000554.6(CRX):c.191del (p.Pro64fs) | ad | LCA | VCV000973905 |  |  |
| NM_000554.6(CRX):c.193G>C (p.Asp65His) | ar, ad, simplex | RP | VCV000143085 | PMID: 18310263, 25324289, 32533067, 37239417 | (Jin et al., 2008, Oishi et al., 2014, Fujinami-Yokokawa et al., 2020, Kim et al., 2023) |
| NM_000554.6(CRX):c.196G>A (p.Val66Ile) | ad | RP, early onset | VCV000099597 | PMID:  11139241, 18055816 | (Sohocki et al., 2001, Vallespin et al., 2007) |
| NM_000554.6(CRX):c.205C>T (p.Arg69Cys) |  | RP | VCV000560444 |  |  |
| NM_000554.6(CRX):c.238G>A (p.Glu80Lys) | ad | CoRD | VCV000099599 | PMID: 10874321, 33691693 | (Sankila et al., 2000, Ma et al., 2021) |
| NM_000554.6(CRX):c.239A>G (p.Glu80Gly) | ad | CoRD | VCV000865803 | PMID: 22960069 | (Huang et al., 2012) |
| NM_000554.6(CRX):c.239A>C (p.Glu80Ala) | ad | CoRD | VCV000007416 | PMID: 1201699, 9390563, 9792858, 11139241 | (Hittner et al., 1975, Freund et al., 1997, Sohocki et al., 1998, Sohocki et al., 2001) |
| NM_000554.6(CRX):c.263A>G (p.Lys88Arg) |  | RP | VCV001020865 |  |  |
| NM_000554.6(CRX):c.G264T(p.Lys88Asn) | ad | LCA |  | PMID: 20513135 | (Nichols et al., 2010) |
| NM_000554.6(CRX):c.262A>G (p.Lys88Glu) |  | LCA |  | PMID: 34653402 | (Chirco et al., 2021) |
| NM_000554.6(CRX):c.268C>T (p.Arg90Trp) | ad, ar | CoRD, LCA | VCV000007422 | PMID: 9931337, 32533067, 32689858 | (Swaroop et al., 1999b, Fujinami-Yokokawa et al., 2020, Ng et al., 2020) |
| NM_000554.6(CRX):c.269G>A (p.Arg90Gln) |  | CoRD | VCV000452003 |  |  |
| NM_000554.6(CRX):c.272G>A (p.Arg90Lys) | ad | MD |  | PMID: 25270190 | (Hull et al., 2014) |
| NM_000554.6(CRX):c.274G>A (p.Ala92Thr) | sporadic | RP and RCD but non-syndromic | VCV000191106 | PMID: 26355662 | (Patel et al., 2016) |
| NM_000554.6(CRX):c.284delG (p.Arg95fs) | simplex | CoRD, putative  pathogenic | | PMID: 26957898 | (Oishi et al., 2016) |
| NM_000554.6(CRX):c.292C>T (p.Arg98Ter) |  | Atypical and mild manifestations | VCV000978221 | PMID: 30460480 | (Zhu et al., 2019) |
| NM_000554.6(CRX):c.295C>T (p.Gln99Ter) |  | MD | VCV000437960 | PMID: 28041643 | (Carss et al., 2017) |
| NM_000554.6(CRX):c.313C>T (p.Gln105Ter) |  | RD | VCV000866673 |  |  |
| NM_000554.6(CRX):c.316C>T (p.Gln106Ter) | ad | CoRD |  | PMID: 33691693 | (Ma et al., 2021) |
| NM_000554.6(CRX):c.335C>T (p.Ala112Val) | ad | CoRD | VCV000099602 | PMID: 22960069 | (Huang et al., 2012) |
| NM_000554.6(CRX):c.344G>A (p.Arg115Gln) | ad | RP | VCV000950982 | PMID: 11139241 | (Sohocki et al., 2001) |
| NM_000554.6(CRX):c.362C>T (p.Ala121Val) | ad | CoRD | VCV002138313 | PMID: 22960069 | (Huang et al., 2012) |
| NM_000554.6(CRX):c.365G>A (p.Gly122Asp) | ad | LCA, rod monochromacy | VCV000099604 | PMID: 11139241, 11748859, 25324289 | (Sohocki et al., 2001, Rivolta et al., 2001b, Oishi et al., 2014) |
| NM_000554.6(CRX):c.378A>C (p.Arg126Ser) |  | RD | VCV000867106 |  |  |
| NM_000554.6(CRX):c.381dup (p.Ser128fs) | ad | Stargart disease | VCV000636020 | PMID: 30718709 | (Jespersgaard et al., 2019) |
| NM_000554.6(CRX):c.380C>T (p.Pro127Leu) |  | inborn genetic diseases | VCV002538604 |  |  |
| NM_000554.6(CRX):c.412A>G (p.Ile138Val) |  | inborn genetic diseases | VCV001349400 |  |  |
| NM_000554.6(CRX):c.413delT (p.Ile138fs) | ad | LCA |  | PMID: 20513135 | (Nichols et al., 2010) |
| NM_000554.6(CRX):c.421delT (p.Ile141fs) | ad | LCA |  | PMID: 24001014 | (Zou et al., 2015) |
| NM_000554.6(CRX):c.425A>G (p.Tyr142Cys) | ad, ar | RP and RCD but non-syndromic, early onset arRP | VCV000099605 | PMID: 18055816, 26355662, 30718709 | (Vallespin et al., 2007, Patel et al., 2016, Jespersgaard et al., 2019) |
| NM_000554.6(CRX):c.429_430delinsA (p.Ser143fs) | ad | LCA |  | PMID: 17964524 | (Stone, 2007) |
| NM_000554.6(CRX):c.429del (p.Pro145fs) | ad | RP |  | PMID: 29847639 | (Martin-Merida et al., 2018) |
| NM_000554.6(CRX):c.430delC (p.Pro145fs) | ad | RP |  | PMID: 32533067 | (Fujinami-Yokokawa et al., 2020) |
| NM_000554.6(CRX):c.431_434del (p.Pro144fs) |  | RD | VCV000866806 |  |  |
| NM_000554.6(CRX):c.431_443del13 (p.Leu146fs) | ad | RP |  | PMID: 24154662 | (Wang et al., 2014a) |
| NM_000554.6(CRX):c.434dup (p.Leu146fs) | ad | CoRD |  | PMID: 29555955 | (Birtel et al., 2018) |
| NM_000554.6(CRX):c.437_449del (p.Leu146fs) |  | RD | VCV000866158 |  |  |
| NM_000554.6(CRX):c.436_447del (p.Leu146_Pro149del) | ad | LCA | VCV000099606 | PMID: 9792858, 11139241 | (Sohocki et al., 1998, Sohocki et al., 2001) |
| NM_000554.6(CRX):c.442delG (p.Gly148fs) | ad | LCA |  | PMID: 28966547 | (Han et al., 2017) |
| NM_000554.6(CRX):c.443del (p.Gly148fs) | ad | LCA | VCV000982533 | PMID: 32165824, 37239417 | (Surl et al., 2020, Kim et al., 2023) |
| NM_000554.6(CRX):c.447dup (p.Ser150fs) | ad | CoRD | VCV000099607 | PMID: 12359607, 25283059 | (Lines et al., 2002, Duncker et al., 2015) |
| NM_000554.6(CRX):c.448_449del (p.Ser150fs) | ad | RP | VCV000437961 | PMID: 28041643 | (Carss et al., 2017) |
| NM_000554.6(CRX):c.449C>G (p.Ser150Ter) | ad | Bull's eye maculopathy | VCV000218921 | PMID: 25259927 | (Yamamoto et al., 2014) |
| NM_000554.6(CRX):c.450del (p.Gly151fs) | ad | LCA | VCV000973941 |  |  |
| NM_000554.6(CRX):c.455C>A (p.Ser152Tyr) | ad | RP |  | PMID: 18310263 | (Jin et al., 2008) |
| NM_000554.6(CRX):c.458del (p.Pro153fs) | ad | LCA | VCV000973942 | PMID:  15994872, 17347810, 21602930, 24093488 | (Ziviello et al., 2005, Wang et al., 2007, Li et al., 2011, Arcot Sadagopan et al., 2015) |
| NM_000554.6(CRX):c.460A>G (p.Thr154Ala) | ad | LCA | VCV000893436 | PMID: 18682808 | (Seong et al., 2008) |
| NM_000554.6(CRX):c.463_464insGGCA (p.Thr155fs) | ad | LCA |  | PMID: 17964524 | (Stone, 2007) |
| NM_000554.6(CRX):c.464C>T (p.Thr155Met) |  | inborn genetic diseases | VCV000859152 |  |  |
| NM_000554.6(CRX):c.472G>A (p.Ala158Thr) | ad | CoRD, LCA | VCV000099608 | PMID: 9427255, 10766140, 11139241, 11748859, 18055820, 20079931 | (Swain et al., 1997, Lotery et al., 2000, Sohocki et al., 2001, Rivolta et al., 2001b, Henderson et al., 2007, Walia et al., 2010) |
| NM_000554.6(CRX):c.480_481del (p.Ser161fs) | ad | LCA | VCV000803570 | PMID: 31630094 | (Xu et al., 2020) |
| NM_000554.6(CRX):c.489G>A (p.Trp163Ter) |  | LCA | VCV000973898 |  |  |
| NM_000554.6(CRX):c.491G>A (p.Ser164Asn) |  | inborn genetic diseases | VCV000859372 |  |  |
| NM_000554.6(CRX):c.495delAinsTTT (p.Ale166fs) | ad | CoRD |  | PMID: 22183351 | (Kohl et al., 2012) |
| NM_000554.6(CRX):c.500_501del (p.Ala166_Ser167insTer) | ad | LCA | VCV000803571 | PMID: 31630094 | (Xu et al., 2020) |
| NM_000554.6(CRX):c.503_504del (p.Glu168fs) | ad | LCA | VCV000099609 | PMID: 9537410, 9804150,10766140, 20079931 | (Freund et al., 1998, Jacobson et al., 1998, Lotery et al., 2000, Walia et al., 2010) |
| NM_000554.6(CRX):c.502del (p.Glu168fs) | ad | CoRD | VCV000007417 | PMID: 9390563 | (Freund et al., 1997) |
| NM_000554.6(CRX):c.509del (p.Pro170fs) | ad | LCA | VCV001359713 | PMID:  12843339, 15024725, 31626798 | (Perrault et al., 2003, Hanein et al., 2004, Yi et al., 2019) |
| NM_000554.6(CRX):c.512del (p.Leu171fs) | ad | LCA | VCV000973899 |  |  |
| NM_000554.6(CRX):c.514delC (p.Pro172fs) | ad | LCA |  | PMID: 28127548 | (Bernardis et al., 2016) |
| NM_000554.6(CRX):c.522_523dup (p.Gln175fs) | ad | LCA | VCV001184468 |  |  |
| NM_000554.6(CRX):c.520del (p.Ala174fs) | ad | LCA | VCV000099611 | PMID: 12208271 | (Nakamura et al., 2002) |
| NM_000554.6(CRX):c.523C>T (p.Gln175Ter) | ad | LCA | VCV000973900 |  |  |
| NM_000554.6(CRX):c.526C>T (p.Arg176Trp) | ad | RCD | VCV001519409 | PMID: 25270190 | (Hull et al., 2014) |
| NM_000554.6(CRX):c.529del (p.Ala177fs) | ad | LCA | VCV000065614 | PMID: 11910559 | (Koenekoop et al., 2002) |
| NM_000554.6(CRX):c.532G>C (p.Gly178Arg) |  | inborn genetic diseases | VCV002221508 |  |  |
| NM_000554.6(CRX):c.533_545dup (p.Gly183fs) | ad | CoRD, MD | VCV001687488 |  |  |
| NM_000554.6(CRX):c.538dupG (p.Val180fs) | ad | CoRD |  | PMID: 30095615 | (Wang et al., 2018) |
| NM_000554.6(CRX):c.541del (p.Ala181fs) | ad | LCA | VCV000099612 | PMID:  11449318, 21602930, 24938718 | (Zhang et al., 2001, Li et al., 2011, Xu et al., 2014) |
| NM_000554.6(CRX):c.543del (p.Ser182fs) | ad | LCA |  | PMID: 27375279 | (Xu et al., 2016) |
| NM_000554.6(CRX):c.545C>G (p.Ser182Ter) | ad | LCA | VCV001685674 | PMID: 31054281, 31630094 | (Gao et al., 2019, Xu et al., 2020) |
| NM_000554.6(CRX):c.549del (p.Pro184fs) | ad | LCA | VCV000973901 |  |  |
| NM_000554.6(CRX):c.557dupT (p.Thr176fs) | ad | CoRD |  | PMID: 31626798 | (Yi et al., 2019) |
| NM_000554.6(CRX):c.564del (p.Ala189fs) | ad | LCA | VCV001172711 |  |  |
| NM_000554.6(CRX):c.564dup (p.Ala189fs) | ad | CoRD |  | PMID: 26103963 | (Boulanger-Scemama et al., 2015) |
| NM_000554.6(CRX):c.570dup (p.Tyr191fs) | ad | LCA, CoRD | VCV001185565 | PMID: 17964524 | (Stone, 2007) |
| NM_000554.6(CRX):c.565G>C (p.Ala189Pro) | ad | CoRD | VCV000894659 |  |  |
| NM_000554.6(CRX):c.565del (p.Ala189fs) | ad | LCA | VCV000599149 |  |  |
| NM_000554.6(CRX):c.566delC (p.Ala189fs) | ad | LCA |  | PMID: 27208204 | (Ellingford et al., 2016a) |
| NM_000554.6(CRX):c.570del (p.Tyr191fs) | ad | LCA | VCV000973902 | PMID: 11748859, 25270190 | (Rivolta et al., 2001b, Hull et al., 2014) |
| NM_000554.6(CRX):c.571del (p.Tyr191fs) | ad | LCA | VCV000099614 | PMID: 11748842, 11748859, 24001014, 25270190 | (Rivolta et al., 2001a, Rivolta et al., 2001b, Hull et al., 2014, Zou et al., 2015) |
| NM_000554.6(CRX):c.573T > G (p.Tyr191Ter) | ad | LCA |  | PMID: 31626798 | (Yi et al., 2019) |
| NM_000554.6(CRX):c.582delC (p.Tyr191fs) | ad | MD |  | PMID: 25270190 | (Hull et al., 2014) |
| NM_000554.6(CRX):c.585dup (p.Ala196fs) | ad | CoRD | VCV000099615 | PMID: 9792858, 11139241 | (Sohocki et al., 1998, Sohocki et al., 2001) |
| NM_000554.6(CRX):c.586_587del (p.Ala196fs) | ad | CoRD | VCV001685675 | PMID: 9427255 | (Swain et al., 1997) |
| NM_000554.6(CRX):c.585C>G (p.Tyr195Ter) | ad | LCA |  | PMID: 27375279 | (Xu et al., 2016) |
| NM_000554.6(CRX):c.585C>A (p.Tyr195Ter) | ad | LCA | VCV000973903 | PMID: 17964524 | (Stone, 2007) |
| NM_000554.6(CRX):c.586del (p.Ala196fs) |  | RD | VCV000867219 |  |  |
| NM_000554.6(CRX):c.586_587insC (p.Ala196fs) | ad | CoRD |  | PMID: 25698705 | (Fernandez-San Jose et al., 2015) |
| NM_000554.6(CRX):c.590del (p.Pro197fs) | ad | Stargardt disease | VCV001399832 | PMID: 29555955, 35934205 | (Birtel et al., 2018, Yahya et al., 2023) |
| NM_000554.6(CRX):c.587delC (p.Pro197fs) | ad | CoRD |  | PMID: 32533067 | (Fujinami-Yokokawa et al., 2020) |
| NM_000554.6(CRX):c.587_590del (p.Ala196fs) | ad | CoRD | VCV000099616 | PMID: 9427255 | (Swain et al., 1997) |
| NM_000554.6(CRX):c.590C>T (p.Pro197Leu) |  | inborn genetic diseases | VCV000965487 |  |  |
| NM_000554.6(CRX):c.590dup (p.Ala198fs) | ad | RP |  | PMID: 29847639 | (Martin-Merida et al., 2018) |
| NM_000554.6(CRX):c.594_606del (p.Ser199fs) |  | RD | VCV000839142 |  |  |
| NM_000554.6(CRX):c.597C>T (p.Ser199=) | ar | RP | VCV000099617 | PMID: 11139241, 11748859 | (Sohocki et al., 2001, Rivolta et al., 2001b) |
| NM_000554.6(CRX):c.605del (p.Cys202fs) | ad | STGD/MD, Macular dystrophy, Cone dystrophy | VCV000236495 | PMID: 25270190, 27208204 | (Hull et al., 2014, Ellingford et al., 2016a) |
| NM_000554.6(CRX):c.608_609del (p.Ser203fs) | ad | CoRD |  | PMID: 26103963 | (Boulanger-Scemama et al., 2015) |
| NM_000554.6(CRX):c.615del (p.Ser206fs) | ad | CoRD | VCV000099618 | PMID: 15531334 | (Itabashi et al., 2004) |
| NM_000554.6(CRX):c.621_623delinsTT (p.Tyr208fs) |  | RD | VCV000866251 |  |  |
| NM_000554.6(CRX):c.624T>G (p.Tyr208Ter) | ad | LCA | VCV001496447 | PMID: 17964524, 20079931, 25270190 | (Stone, 2007, Walia et al., 2010, Hull et al., 2014) |
| NM_000554.6(CRX):c.636delC (p.Ser213fs) | ad | CoRD |  | PMID: 18653602, 22183351, 31626798 | (Kitiratschky et al., 2008, Kohl et al., 2012, Yi et al., 2019) |
| NM_000554.6(CRX):c.642T > G (p.Tyr214Ter) | ad | LCA |  | PMID: 31626798 | (Yi et al., 2019) |
| NM_000554.6(CRX):c.648del (p.Ser216fs) | ad | RP, LCA | VCV000224738 | PMID: 26872967 | (Ellingford et al., 2016b) |
| NM_000554.6(CRX):c.649G>A (p.Gly217Ser) |  | inborn genetic diseases | VCV001369314 |  |  |
| NM_000554.6(CRX):c.650del (p.Gly217fs) | ad | LCA | VCV000099619 | PMID: 9537410, 10766140, 31626798 | (Freund et al., 1998, Lotery et al., 2000, Yi et al., 2019) |
| NM_000554.6(CRX):unavailable (p.Asp219fs) | ad | Bull's eye maculopathy |  | PMID: 25259927 | (Yamamoto et al., 2014) |
| NM_000554.6(CRX):c.660del (p.Tyr221fs) | ad | Bull's eye maculopathy | VCV000218922 | PMID: 25259927 | (Yamamoto et al., 2014) |
| NM_000554.6(CRX):c.663C>G (p.Tyr221Ter) | ad | LCA | VCV002152320 | PMID: 31630094 | (Xu et al., 2020) |
| NM_000554.6(CRX):c.663C>A (p.Tyr221Ter) | ad | CoRD, RD | VCV000425193 | PMID: 29555955 | (Birtel et al., 2018) |
| NM_000554.6(CRX):c.682C>T (p.Gln228Ter) | ad | RP |  | PMID: 26667666 | (Ge et al., 2015) |
| NM_000554.6(CRX):c.684_685delinsAA (p.Leu229Ile) |  | RD | VCV000866884 |  |  |
| NM_000554.6(CRX):c.684G>C (p.Gln228His) | ad | RP | VCV000636021 | PMID: 30718709, 37239417 | (Jespersgaard et al., 2019, Kim et al., 2023) |
| NM_000554.6(CRX):c.685C>A (p.Leu229Ile) |  | inborn genetic diseases | VCV002217502 |  |  |
| NM_000554.6(CRX):c.692delG (p.Gly231fs) | ad | LCA |  | PMID: 31626798 | (Yi et al., 2019) |
| NM_000554.6(CRX):c.695del (p.Pro232fs) | ad | LCA, RP, RP and RCD but non-syndromic | VCV000191298 | PMID: 26355662, elongating mutation | (Patel et al., 2016) |
| NM_000554.6(CRX):c.709del (p.Leu237fs) | ad | LCA | VCV000099620 | PMID: 10892846, 11748842, 18055820 | (Silva et al., 2000) |
| NM_000554.6(CRX):c.709dupC (p.Leu237fs) | ad | LCA |  | PMID: 17964524 | (Stone, 2007) |
| NM_000554.6(CRX):c.714del (p.Gly239fs) |  |  | VCV002033764 | elongating mutation |  |
| NM_000554.6(CRX):c.724G>A (p.Val242Met) | ad | CoRD, RP | VCV000099621 | PMID: 9427255, 11748859 | (Swain et al., 1997, Rivolta et al., 2001b) |
| NM_000554.6(CRX):c.747delC (p.Ser249fs) | ad | LCA |  | PMID: 20079931 | (Walia et al., 2010) |
| NM_000554.6(CRX):c.750del (p.Thr251fs) |  |  | VCV001418367 | elongating mutation |  |
| NM_000554.6(CRX):c.753del (p.Ser252fs) |  |  | VCV000099622 | elongating mutation |  |
| NM_000554.6(CRX):c.766C>T (p.Gln256Ter) | ad | CoRD | VCV000861103 | PMID: 26682157, 28945142 | (Lu et al., 2015, Griffith et al., 2018) |
| NM_000554.6(CRX):c.774T>G (p.Tyr258Ter) | ad | MD |  | PMID: 22968130 | (Shanks et al., 2013) |
| NM_000554.6(CRX):c.774T>A (p.Tyr258Ter) | ad | CoRD, MD | VCV001479901 | PMID:  22968130, 25270190 | (Shanks et al., 2013, Hull et al., 2014) |
| NM_000554.6(CRX):c.782delA (p.Tyr261fs) | ad | LCA |  | PMID: 31630094 | (Xu et al., 2020) |
| NM_000554.6(CRX):c.785delC (p.Val264fs) | ad | LCA |  | PMID: 28181551 | (Riera et al., 2017) |
| NM_000554.6(CRX):c.787delC (p.Val264fs) | ad | LCA |  | PMID: 11748859, 20079931 | (Rivolta et al., 2001b, Walia et al., 2010) |
| NM_000554.6(CRX):c.787_790del (p.Pro263fs) | ad | LCA |  | PMID: 31626798 | (Yi et al., 2019) |
| NM_000554.6(CRX):c.790G>A (p.Val264Met) | ad, sporadic | CoRD | VCV001465917 | PMID: 25356976, 31626798 | (Huang et al., 2015, Yi et al., 2019) |
| NM_000554.6(CRX):c.798del (p.Leu267fs) |  |  | VCV001053714 | elongating mutation |  |
| NM_000554.6(CRX):c.806_809dup (p.Lys270fs) |  |  | VCV001059600 | elongating mutation |  |
| NM_000554.6(CRX):c.816del (p.Thr273fs) | ad | LCA | VCV000973904 | elongating mutation |  |
| NM_000554.6(CRX):c.816_818delinsAA (p.Thr273fs) | ad | CoRD | VCV000007425 | PMID: 17320181 | (Paunescu et al., 2007) |
| NM_000554.6(CRX):c.818C>T (p.Thr273Met) | ad | LCA | VCV000099624 | PMID: 16123401 | (Zernant et al., 2005) |
| NM_000554.6(CRX):c.821delG (p.Gly274fs) | ad | CoRD |  | PMID: 25270190 | (Hull et al., 2014) |
| NM_000554.6(CRX):c.827G>A (p.Trp276Ter) | ad | Stargardt disease | VCV000636022 | PMID: 30718709 | (Jespersgaard et al., 2019) |
| NM_000554.6(CRX):c.856_865delinsGATCCC (p.Leu286fs) | ad |  | VCV000497549 | elongating mutation |  |
| NM_000554.6(CRX):c.887T>G (p.Phe296Cys) | ad | CoRD |  | PMID: 17525851 | (Preising et al., 2007) |
| NM_000554.6(CRX):c.897G>C (p.Leu299Phe) | ad | RP | VCV000143086 | PMID: 25324289 | (Oishi et al., 2014) |
| NM_000554.6(CRX):c.898T>C (p.Ter300Gln) | ad (sporadic) | CoRD | VCV000829982 | PMID: 37239417 | (Kim et al., 2023) |
| NM_000554.6(CRX):c.899A>G (p.Ter300Gln) | ad | CoRD |  | PMID: 17525851 | (Preising et al., 2007) |
|  |  |  |  |  |  |
| **Copy number variants** |  |  |  |  |  |
| NC_000019.9:g.(?_48339480)_(48343224_?)del | ad | LCA | VCV002423325 | PMID: 27032803, 29847639, 31743059 | (Bravo-Gil et al., 2016, Martin-Merida et al., 2018, Khan et al., 2019) |
| NM_000554.4:c.(?_-1)_(*1_?)dup | ad | RP | VCV000636230 | PMID: 30718709 | (Jespersgaard et al., 2019) |
| GRCh38/hg38 19q13.32-13.33(chr19:44971420-48257402)x3 |  | RD | VCV000147383 | PMID: 20466091 | (Miller et al., 2010) |
| GRCh38/hg38 19q13.32-13.33(chr19:46658791-49050450)x3 | ad | RD | VCV000059115 | PMID: 21844811 | (Kaminsky et al., 2011) |
| GRCh38/hg38 19q13.32-13.33(chr19:47257435-47886413)x1 | ad | CoRD | VCV002579250 | PMID: 29555955 | (Birtel et al., 2018) |
| GRCh38/hg38 19q13.33(chr19:47794370-47886413)x1 | ad | CoRD | VCV002579248 | PMID: 29555955 | (Birtel et al., 2018) |
| Single allele | ad, maternally inherited, no vision concerns | mild intellectual disability, speech and language disorder, and ADHD | VCV000560119 |  |  |
| GRCh38/hg38 19q13.33(chr19:47832818-47902680)x3 |  | RD | VCV000148447 | PMID: 20466091 | (Miller et al., 2010) |
| GRCh38/hg38 19q13.33(chr19:47834312-47840923)x1 | ad | CoRD | VCV002579249 | PMID: 29555955 | (Birtel et al., 2018) |
| GRCh37/hg19 (chr19:48305033-48389514)x1 | ad | MD |  | PMID: 35934205 | (Yahya et al., 2023) |
| GRCh37/hg19 (chr19:48337702-48343224)x1 | ad | MD |  | PMID: 35934205 | (Yahya et al., 2023) |
| GRCh37/hg19 (chr19:48407000-48465000)x1 | ad | MD |  | PMID: 35934205 | (Yahya et al., 2023) |
| GRCh37/hg19 (chr19:48337702-48389514)x1 | ad | MD |  | PMID: 35934205 | (Yahya et al., 2023) |
|  |  |  |  |  |  |
| **Intronic variants** |  |  |  |  |  |
| NM_000554.6(CRX):c.100+1G>C |  | STGD/MD | VCV000236496 | PMID: 27208204 | (Ellingford et al., 2016a) |
| NM_000554.6(CRX):c.101-1G>A |  |  | VCV000983150 | PMID:  32165824, 37239417 | (Surl et al., 2020, Kim et al., 2023) |
| NM_000554.6(CRX):c.252+1G>A | ad | CoRD |  | PMID: 26103963 | (Boulanger-Scemama et al., 2015) |
