# Supplementary material for: Transcriptional precision in photoreceptor development and diseases – Lessons from 25 years of CRX research
Source: Front Cell Neurosci. 2024 Feb 13;18:1347436. doi: 10.3389/fncel.2024.1347436 (PMC10896975; doi:10.3389/fncel.2024.1347436)
Supplement: Supplementary file 2 [file Table_2.docx]

**Supplementary Table S2.** Retinal phenotype summary of *Crx* variant knock-in mouse models

† ad: autosomal dominant; ar: autosomal recessive; LCA: Leber congenital amaurosis; CoRD: Cone rod dystrophy. TVRM65 and RIP are spontaneous variants found in mice, thus no human disease phenotype is provided.

* All phenotypes are given in the C57BL/6J background.

“+” qualitative graded values; “-“ null or not significant; “ND” not determined.

| Variant (Disease)^†^ | Pathogenic class | Mouse model^*^ | Function  (ERG at 1month) | | OS length | Degeneration time course | | Gene expression (compared to P10) | | Publication |
| --- | --- | --- | --- | --- | --- | --- | --- | --- | --- | --- |
|  |  |  | Rod | Cone | Rod | Rod | Cone | P10 | P21 |  |
|  |  | WT | ++++ | ++++ | +++++ | - | - | ++ | +++++ | (Tran et al., 2014, Ruzycki et al., 2015) |
|  |  | *+/-* | ++++ | ++++ | +++++ | - | - | ++ | +++++ | (Tran et al., 2014, Ruzycki et al., 2015) |
|  |  | *-/-* | - | - | - | 1-3 months | 1-3 months | - | - |  |
| E168d2  (adLCA) | Truncated  C-terminus | *E168d2/+* | + | + | ++ | 1-6 months | 1 month | + | + | (Tran et al., 2014, Ruzycki et al., 2015) |
|  |  | *E168d2/d2* | - | - | - | 1-3 months | 1-3 months | - | - |  |
| TVRM65 | Truncated  C-terminus | *L253X/+* | +++ | +++ | +++++ | - | - | + | +++ | (Ruzycki et al., 2017) |
|  |  | *L253X/X* | - | - | - | 1-3 months | 1-3 months | - | - |  |
| RIP | Extended  C-terminus | *Rip/+* | - | - | - | Thinner ONL but preserved  >= 18months | | ND | - | (Roger et al., 2014) |
|  |  | *Rip/Rip* | - | - | - | 1-9 months | 1-9 months | ND | - |  |
| R90W  (arLCA) | Reduced DNA binding affinity | *R90W/+* | ++++ | ++++ | +++++ | - | - | ++ | +++++ | (Tran et al., 2014, Ruzycki et al., 2015) |
|  |  | *R90W/W* | - | - | - | 1-3 months | 1-3 months | - | - |  |
| E80A  (adCoRD) | Altered DNA binding specificity | *E80A/+* | ++ | - | +++ | - | - | +++ | +++ | (Zheng et al., 2023) |
|  |  | *E80A/A* | - | - | + | - | - | +++ | + |  |
| K88N  (adLCA) | Altered DNA binding specificity | *K88N/+* | - | - | - | - | - | - | - | (Zheng et al., 2023) |
|  |  | *K88N/N* | - | - | - | - | - | - | - |  |
